# Transcription phase, protein characteristics of DEV UL45 and prokaryotic expression, antibody preparation of the UL45 des-transmembrane domain

**DOI:** 10.1186/1743-422X-7-232

**Published:** 2010-09-16

**Authors:** Ai-Mei Shen, Guang-Peng Ma, An-Chun Cheng, Ming-Shu Wang, Dan-Dan Luo, Li-Ting Lu, Tao Zhou, De-Kang Zhu, Qi-Hui Luo, Ren-Yong Jia, Zheng-Li Chen, Yi Zhou, Xiao-Yue Chen

**Affiliations:** 1 Avian Diseases Research Center, College of Veterinary Medicine of Sichuan Agricultural University, Ya'an 625014, Sichuan China; 2 Key Laboratory of Animal Diseases and Human Health of Sichuan Province, Ya'an 625014, Sichuan Province, China; 3 Epizootic Diseases Institute of Sichuan Agricultural University, Ya'an, China; 4 China Rural Technology Development Center, Beijing, 100045, China

## Abstract

**Background:**

Some UL45 gene function of Herpesvirus was reported. While there was no any report of the duck enteritis virus (DEV) UL45 protein as yet.

**Results:**

The UL45 gene and des-transmembrane domain of UL45 (named UL45Δ gene, 295-675bp of UL45) of DEV were amplified by PCR and subcloned into the prokaryotic expression vector pET-32a(+). The constructed recombinant plasmids were transformed into the host strain BL21(DE3) PLysS and induced by IPTG. SDS-PAGE analysis showed the UL45 gene couldn't express while UL45Δ gene was highly expressed. His Purify Kit or salting-out could purify the protein effectively. Using the purified protein to immunize New-Zealand rabbits and produce polyclonal antibody. The agar diffusion reaction showed the titer of antibody was 1:32. Western blot analysis indicated the purified rabbit anti-UL45Δ IgG had a high level of specificity and the UL45 gene was a part of DEV genome. The transcription phase study of UL45 gene showed that expression of UL45 mRNA was at a low level from 0 to 18 h post-infection (pi), then accumulated quickly at 24 h pi and peaked at 42 h pi. It can be detected till 72 h pi. Besides, western blot analysis of purified virion and different viral ingredients showed that the UL45 protein resided in the purified virion and the viral envelope.

**Conclusions:**

The rabbit anti-UL45Δ IgG was produced successfully and it can serve as a good tool for penetrating studies of the function of DEV UL45 protein. The transcription phase and protein characteristics analysis indicated that DEV UL45 gene was a late gene and UL45 protein may be a viral envelope protein.

## Background

Duck virus enteritis (DVE) or duck plague (DP), was an acute, febrile, contagious and septic disease of waterfowl (duck, goose, and swan) caused by Duck Enteritis Virus (DEV). It caused considerable economic losses to the duck-producing areas of the world due to its high mortality and decreased egg production [1-5]. DEV was currently classified to the Alphaherpesvirinae subfamily of the Herpesviridae, but had not been grouped into any genus yet [6]. For a long time, studies of the molecular biology of DEV had larged behind other members of the herpesviridae family. Luckily, during the past several years, some DEV genes had been reported successfully [7-27]. However, the function of potential proteins encoded by many of the DEV genes was still unclear, including UL45.

The conservatism of UL45 gene was low in different herpervirus subfamily, while in different strains of the same herpervirus it was highly conserved [28-31]. The UL45 protein was a true late protein and a component of the virion from other herpesvirinae [32-34]. The main function of UL45 protein which had reported included promoting the cell-cell fusion, anti-apoptosis, viral correct propagation, egress and keeping virulence of virus [35-37].

Using a series of software to analyze the bioinformatics of DEV UL45 gene, the results indicated that UL45 protein had 224 residues with a molecular mass of 24kDa, 73 to 95 amino acids was a potential membrane-spanning segment and no cleavage site of signal peptide. When the threshold was defined to 0.5, it had 13 potential phosphorylation sites and no glycosylation site.

In this article, the construction of cloning and expression plasmids, expression of UL45Δ fusion protein, production of polyclonal antibody, time course of transcription and protein characteristics analysis were detailedly introduced.

## Results

### Gene amplification, construct expression plasmids

Using genome of DEV CHv-strain to amplify the UL45 and UL45Δ gene. Electrophoresis analysis results of amplified products showed that the size of UL45 and UL45Δ gene was the same as expected.

**Figure 1:**
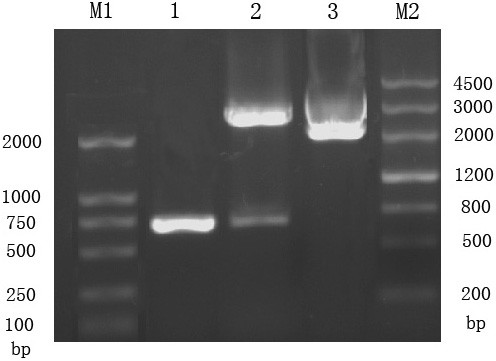
**Identification of recombinant plasmid pMD18-T/UL45 by restriction enzyme digestion and PCR**. M1: DL2000 DNA Marker; M2: MarkerIII DNA Marker; 1: PCR product from pMD18-T/UL45; 2: Product from pMD18-T/UL45 digested by *BamHI *and *XHoI *; 3: The pMD18-T/UL45 plasmids.

**Figure 2:**
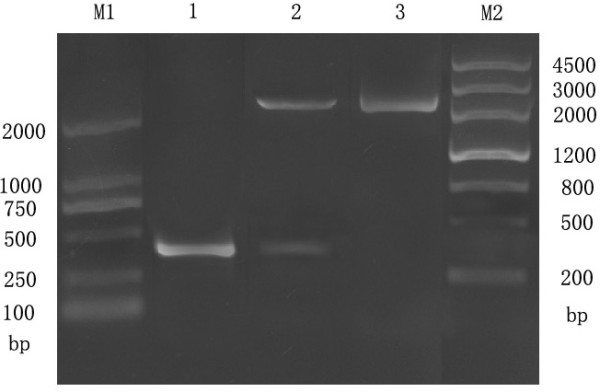
**Identification of recombinant plasmid pMD18-T/UL45Δ by restriction enzyme digestion and PCR**. M1: DL2000 DNA Marker; M2: MarkerIII DNA Marker; 1: PCR product from pMD18-T/UL45Δ; 2: Product from pMD18-T/UL45Δ digested by *BamHI *and *XHoI *; 3: The pMD18-T/UL45Δ plasmids.

**Figure 3:**
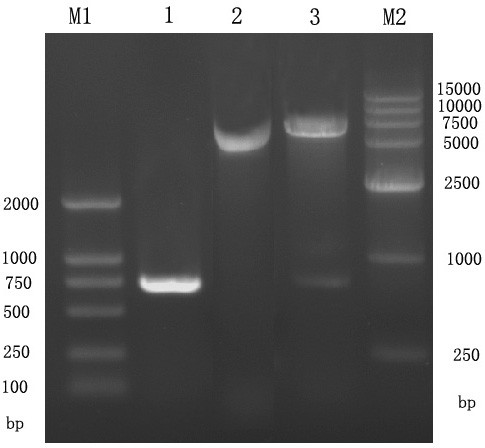
**Identification of recombinant plasmid PET32a(+)-UL45 by restriction enzyme digestion and PCR**. M1: DL2000 DNA Marker; M2: DL15000 DNA Marker; 1: PCR product from PET32a(+)-UL45; 2: PET32a(+)-UL45 plasmids; 3: Product from PET32a(+)-UL45 plasmids digested by *BamHI *and *XHoI *.

**Figure 4:**
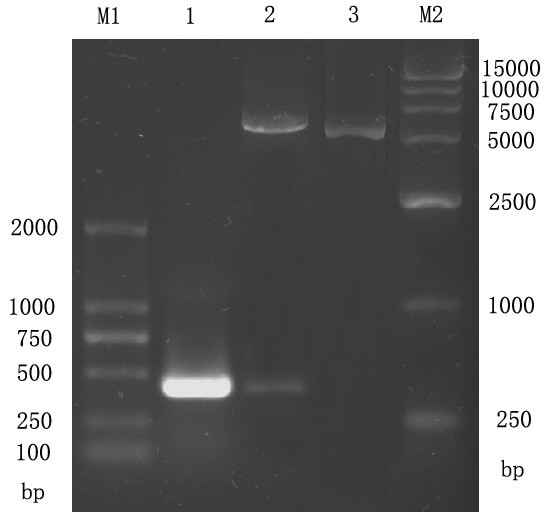
**Identification of recombinant plasmid PET32a(+)-UL45Δ by restriction enzyme digestion and PCR**. M1: DL2000 DNA Marker; M2: DL15000 DNA Marker; 1: PCR product from PET32a(+)-UL45Δ; 2: Product from PET32a(+)-UL45Δ plasmids digested by *BamHI *and *XHoI *; 3: PET32a(+)-UL45Δ plasmids.

The UL45 and UL45Δ gene digested from pMD18-T/UL45 and PMD18-T/UL45Δ plasmids (constructed by TaKaRa) were respectively directionally inserted to pET-32a(+) plasmid to construct the expression plasmids (fig. 1, 2). PCR and restriction digestion analysis showed the UL45 and UL45Δ expression plasmids were successfully constructed (fig. 3, 4).

### Protein expression, purification, polyclone antibody production and western blot analysis

**Figure 5:**
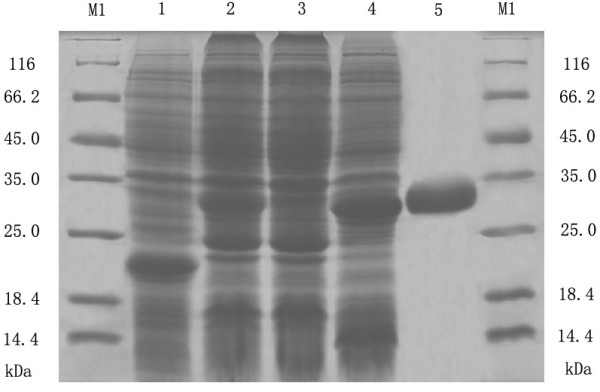
**Expression and purification of recombinant protein**. M1: Protein Marker; 1: Total protein from pET-32a (+) after induction; 2: Total protein from recombinant plasmid PET32a(+)-UL45Δ after induction; 3: Uninduced control; 4: The clear supernatant after ultrasonic disruption; 5: Purified recombinant UL45Δ protein using a single chromatographic step of IMAC on Ni ^2+ ^-NTA agarose.

**Figure 6:**
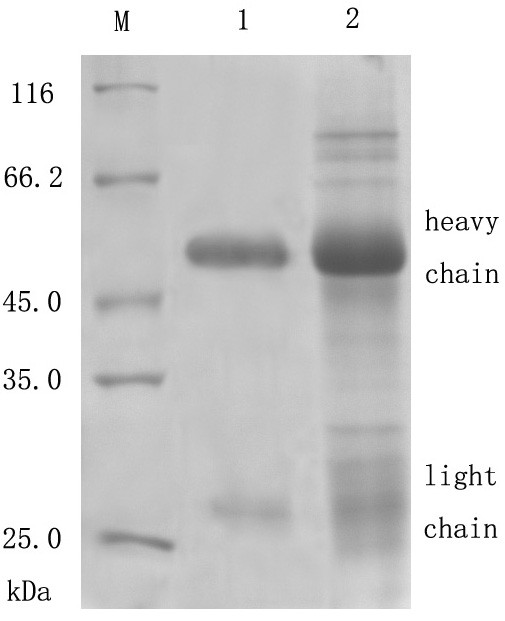
**Purified rabbit anti-UL45Δ IgG**. M: Protein molecular weight marker; 1: Rabbit anti-UL45Δ IgG obtained by ionexchange column chromatography; 2: Rabbit anti-UL45Δ IgG obtained by ammonium sulfate precipitation.

**Figure 7:**
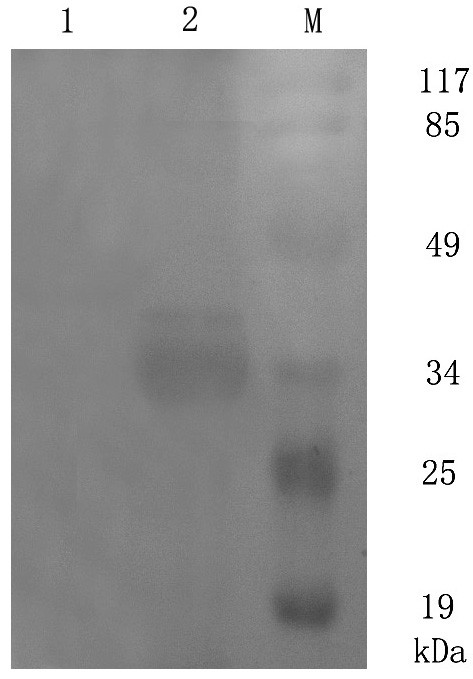
**Identification of the purified recombinant proteins with rabbit anti-UL45Δ IgG by Western-blotting**. M: Prestained protein marker; 1: Negative control serum reacted with UL45Δ protein; 2: Rabbit anti-UL45Δ IgG reacted with UL45Δ protein.

**Figure 8:**
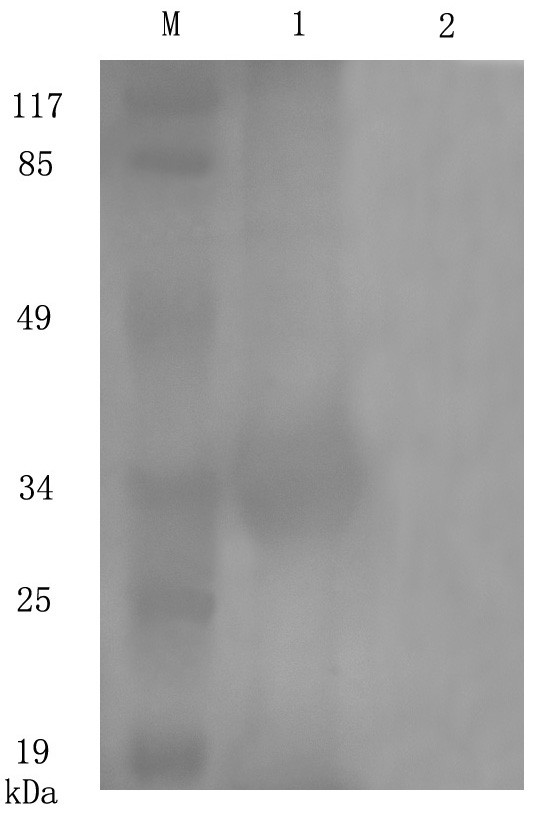
**Identification of the purified recombinant proteins with rabbit anti-DEV IgG by Western-blotting**. M: Prestained protein marker; 1: Rabbit anti-DEV IgG reacted with UL45Δ protein; 2: Negative control serum reacted with UL45Δ protein.

The protein expression condition was analyzed by SDS-PAGE. It showed that the UL45 gene couldn't express while UL45Δ gene was highly expressed in the supernatant. The optimized condition of expression was inducing 4 h at 30°C after adding 0.2 mmol/L IPTG. The UL45Δ protein could be purified effectively by IMAC on Ni^2+^-NTA agarose or salting-out (fig. 5). Using the protein to immune rabbits, after four injections the rabbit anti-UL45Δ serum was collected. Agar diffusion reaction showed the titer of antibody was 1:32. With the methods of ammonium sulfate precipitation and DEAE-Sepharose column, we got the homogeneous rabbit anti-UL45Δ IgG (fig. 6). Importantly, western blot analysis showed that the UL45Δ protein could be recognized by the rabbit anti-UL45Δ IgG and rabbit anti-DEV IgG but it couldn't be recognized by the negative control serum (fig. 7, 8). These showed that the rabbit anti-UL45Δ IgG had a good specificity and UL45 gene was a member of DEV genome.

### Transcription characteristics analysis of UL45 gene

**Figure 9:**
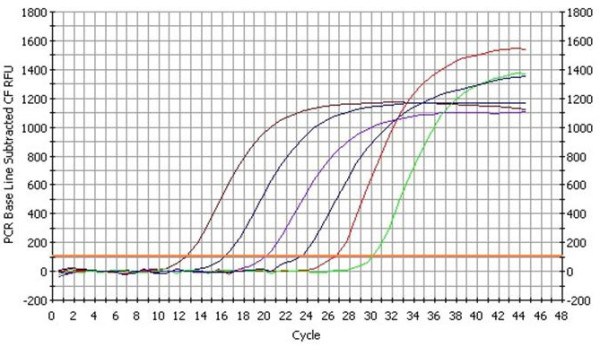
**The fluorescent quantitative real-time PCR amplification curve of β-actin**. The amplification graph of β-actin was composed of six strip almost isometric S-type curves. The curves represented PMD18-T/β-actin plasmids of 10^-3^, 10^-4^, 10^-5^, 10^-6^, 10^-7^and10^-8 ^dilution.

**Figure 10:**
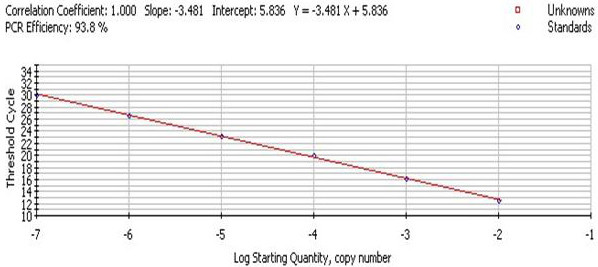
**The fluorescent quantitative real-time PCR standard curve of β-actin**. The x-axis represented ten-fold dilutions of PMD18-T/β-actin plasmids, and the y-axis represented corresponding cycle threshold (*Ct*) values. Each dot represents the result of triplicate amplification of each dilution. The standard curve equation is Y = -3.481 × + 5.836, the correlation coefficient and the slope value of the regression curve were indicated in the figure.

**Figure 11:**
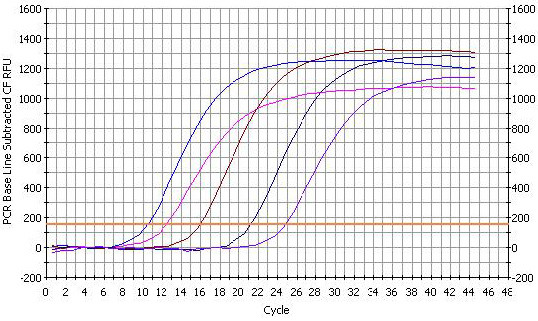
**The fluorescent quantitative real-time PCR amplification curve of UL45**. The amplification graph of UL45 was composed of five strip almost isometric S-type curves. The curves represented PMD18-T/UL45 plasmids of 10^-2^, 10^-3^, 10^-4^, 10^-5 ^and 10^-6^dilution.

**Figure 12:**
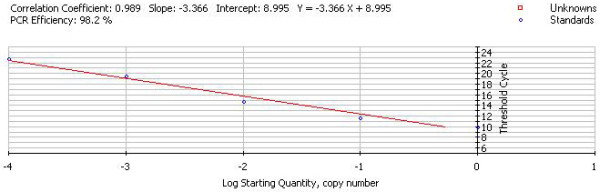
**The fluorescent quantitative real-time PCR standard curve of UL45**. The x-axis represented ten-fold dilutions of PMD18-T/UL45 plasmids, and the y-axis represented corresponding cycle threshold (*Ct*) values. Each dot represents the result of triplicate amplification of each dilution. The standard curve equation is Y = -3.366 × +8.995. The correlation coefficient and the slope value of the regression curve were indicated in the figure.

**Figure 13:**
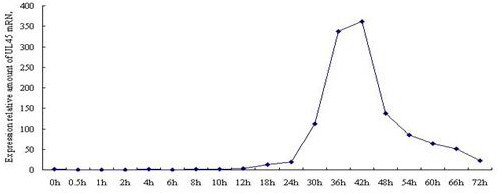
**The transcription level diagram of UL45 gene at different time of infection DEV**.

The standard curve of PMD18-T/UL45 and PMD18-T/β-actin showed the FQ-PCR was excellent at performance (fig. 9, 10, 11 and 12). The integrality and purity detecting of total RNA showed that OD260/OD280 was from 1.8 to 2.0, and the 28 S, 18 S and 5 S could be clearly seen by agarose gel electrophoresis. This indicated that the RNA could be used for further study. The condition of UL45 mRNA expression showed that the situation of transcription was changed during the whole cycle. The expression of DEV UL45 mRNA was at a low level from 0 to 18 h post-infection (pi), then accumulated quickly at 24 h pi and peaked at 42 h pi. It can be detected till 72 h pi (Fig. 13). This inferred that the UL45 gene of DEV-CHv was a late gene.

### Characteristics analysis of UL45 protein

**Figure 14:**
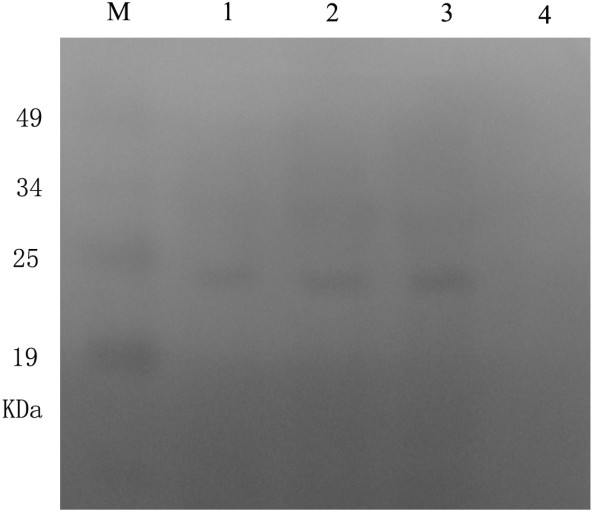
**Western-blot analysis of purified virion and fraction of virion**. M: Prestained protein marker; 1, 2: The supernatant extracted the virion with NP-40 detergent; 3: Purified virion; 4: The pellet extracted the virion with NP-40 detergent.

When analyzing the purified virion with the rabbit anti-UL45Δ IgG, the UL45 protein could be detected and this suggested that the UL45 protein was a component of virion. We extracted the virion with NP-40 detergent, and obtained a supernatant fraction (envelope and minor amounts of tegument proteins) and a pellet (nucleocapsids and tegument proteins). Equivalent amounts of the supernatant and pellet proteins were immunoblotted with the UL45Δ IgG. The results showed that the 24kDa UL45 protein was found almost exclusively in the NP-40 soluble fract, suggested that the UL45 protein was associated with the envelope of the virion (Fig. 14).

## Discussion

Choosing the pET-32a(+), the *E.coli *strain DH5α and *E.coli *strain BL21(DE3) PlysS as the expression vector, cloning and expression host because of their unexampled advantages. Here, *E. coli *strain DH5α has very high transformation efficiency. The *E. coli *strain BL21(DE3) PlysS has the advantage of being deficient in both the lon and ompT proteases and harboring the T7 bacteriophage RNA polymerase gene which permits the specific expression of heterologous genes driven by the T7 promoter [38-40]. Prokaryotic expression vector pET-32a(+), which features a high stringency T7 lac promoter, His6 tag and T7 terminator, has been recognized as one of the most powerful tools for producing recombinant proteins in *E. coli *[41]. This fusion tag permits purification of the produced protein by metal chelate chromatography on a nitrilo-triacetic acid agarose matrix charged with nickel ions. Slight inorganic salt (ammonium sulfate, sodium sulfate etc) can promote the dissolution of proteins but fortis saline solution will induce proteins separated from the solution because of agglomeration, and this effect is called salting-out. The solubility of proteins in aqueous solution is determinated by the extent of hydration between the hydrophilic grouping of proteins and water, the situation of the protein electric charge. Adding the neutral salt to the protein solution, the hydration shell around the proteins becomes weaken or vanished, and the surface charge of protein is neutralized greatly. These lead to the depression of the proteins solubility and separation from the solution. Salting-out is a good method to purify the dissolvable proteins.

Here, the UL45 protein couldn't express regardless of the expression vector, expression host strain and the condition of expression. The possible reasons were as follows. First, the codon could influence the expression of protein. AGA, AGG, AUA, CCG, CCT, CTC, CGA, GTC etc codon was the rare codon of *E.coli *[42,43]. If the exogenous gene had a high level of rare codon then the efficiency of expression was usually low. The statistics of the UL45 gene codon usage condition showed that rare codon had a high usage frequency in UL45 gene, and this may be a reason of the UL45 gene expression failure. Besides, the bioinformatics analysis showed that the 73 to 95 amino acids of UL45 protein was a membrane-spanning segment. As we know, the membrane-spanning segment was high hydrophobic and not good for protein expression. We designed a couple of primers to amplify the UL45Δ gene (deletion of the membrane-spanning segment) and it was expressed with high performance. So we inferred that the existence of membrane-spanning segment was the main reason of the UL45 gene expression failure.

There had been some function studies of UL45 protein from other herpesvirus but there wasn't any report about the function of DEV UL45 protein till now. We used the rabbit anti-UL45Δ IgG to study the DEV UL45 gene transcription phase and the UL45 protein characteristics. These may supply effective evidence to explain the function of UL45 protein in the DEV infection, replication of life cycle.

According to the different transcription sequence, gene can be classified as immediate early gene, early gene and late gene [44]. After the virus infected the target cell, linear double strands DNA which locates in intra-nuclear becomes cyclization, and the virus gene starts to transcript according to some sequence. The late gene encodes about thirty viral proteins, primarily including capsid protein, tegument protein and envelope protein. They usually express at last [45]. Up to now, there wasn't any report of transcription characterization about DEV UL45 gene, while the situation of transcription could reflect the viral genetic structure and the basic situation of gene expression. So the transcription course of UL45 gene was studied, and the results showed that DEV UL45 gene may be a late gene. It was consistent with the report of other herpesvirus and it could provide guidance for future study [28,33]. While only using the FQ-PCR to affirm the UL45 gene is a late gene isn't sufficient, more penetrating studies need to be done. The UL45 gene temporal regulation condition should be analyzed when the infected cell is dealt with some canonical medicine. Such as γ gene can't be detected in the cell dealt with phosphonoacetate; α gene can be detected in the cell dealt with protein synthesis inhibitor; β gene can be inhibited by cycloheximide (protein synthesis inhibitor) while can't be inhibited by phosphonoacetate (DNA synthesis inhibition factor) [46].

The protein characteristics analysis showed that UL45 protein was related with the virion and may be an envelope protein. The UL45 protein may not be a tegument protein, because most tegument protein resided in the sediment and there was no positive signal can be detected in the sediment. However, we couldn't exclude the possibility of UL45 protein was noncohesive binding with the tegument. The result of protein characteristics analysis was coincidence with the transcription characteristics. They all showed that the DEV UL45 gene was a late gene. While the affirmation of UL45 protein characteristics needs more penetrating studies, such as using the protein which had known the characteristics do the parallel experiment or using the ^35^S mark the methionine to confirm the effect of virion purification and so on.

## Conclusions

In conclusions, the UL45Δ protein and rabbit anti-UL45Δ IgG were produced successfully. Western blot analysis showed that the purified UL45Δ protein could be recognized by the rabbit anti-UL45Δ IgG and rabbit anti-DEV IgG, indicating that this specific antibody could serve as a good tool for penetrating studies of DEV UL45 protein function. The transcription phase and protein characteristics analysis indicated that UL45 gene of DEV was a late gene and UL45 protein may be associated with viral envelope. But we still need to do more penetrating studies to ascertain these conclusions.

## Materials and methods

### Virus, strains, vector and main reagents

DEV CHv-strain (a high-virulence field strain of DEV. Separated, identified and preserved by the author's laboratory); *E. coli *strain DH5α, *E. coli *BL21(DE3) PlysS and expression vector pET-32a(+) were preserved in the author's laboratory; Restriction enzymes and ligase mixture were purchased from TaKaRa.

PrimeSTAR HS (Premix) DNA polymerase, DNA and protein molecular weight markers, TIANpure Mini plasmid Kit (catalogue No: DP104-02), TIANgel Midi purification Kit (catalogue No: DP209-03), RNAprep Pure Cell/Bacteria Kit (catalogue No: DP430), Quant Reverse Transcriptase (catalogue No: ER103-04) were purchased from Tiangen Biotech Company; Horseradish peroxidase (HRP)-labeled goat anti-rabbit IgG was purchased from Zhongshan goldenbridge Biotechnology Company; Rabbit anti-DEV IgG was provided by the author's laboratory.

### PCR amplification of the DEV UL45 and UL45Δ gene

Using 11 daytime duck-embryo (purchased from nonimmune region in Ya'an) to prepare the duck embryo fibroblast (DEF) [47]. 24 h later, inculated the DEV-CHv. When the cytopathic effect (CPE) reached 80% harvested the DEF. After three freeze-thaw cycles, using the method of phenol-chloroform to extracted DEV DNA [48].

Considering the order of UL45 (GenBank accession no: EU195107, not release yet), the primers for amplification of UL45 and UL45Δ gene were designed using biological software Oligo6.0 and synthesized by TaKaRa. The forward primers of UL45 and UL45Δ (F1/F2) were 5'-GGATCCCGGATCACCCTAACAATG-3' and 5'-GGATCCACTACAGCGTGGGATACGA-3', the same reverse primer (R1) was 5'-CTCGAGAAACACGCATACAAATAACAAGTC -3', containing the *BamHI *and *XHo I *restriction sites (underlined), respectively. Using the genome of DEV CHv strain as the template, the PCR reactions (50 μl/tube) containing 25 μl PrimeSTAR HS (Premix) DNA polymerase, 1.4 μl of each primer (10pmol each), 2.6 μl DNA template and 19.6 μl ultrapure water was performed. The condition of PCR amplification was initial denaturation at 94°C for 5 min followed by 30 consecutive cycles of denaturation at 94°C for 60 s, annealing at 60.5°C for 60 s, and extension at 72°C for 60 s, and then a final extension at 72°C for 10 min. The amplified products were analyzed by electrophoresis on a 1.2% (w/v) agarose gel.

### Construct the expression plasmids PET32a(+)/UL45 and PET32a(+)/UL45Δ

The PCR products of UL45 and UL45Δ gene were sent to TaKaRa for constructing cloning plasmids. The pMD18-T/UL45 and pMD18-T/UL45Δ plasmids were confirmed by sequencing. The UL45 gene digested from pMD18-T/UL45 (retrieving by TIANgel Midi purification Kit) was directionally ligated into the previously *BamHI */*XHcI *-digested expression vector pET-32a(+). The ligation mixture was transformed into competent *E. coli *DH5α cells for storing. The positive colony was identified by PCR and restriction analysis. Extracted positive plasmids and transformed into competent *E. coli *BL21 (DE3) PlysS strain. Do the same operation to the cloning plasmids pMD18-T/UL45Δ.

### Protein expression, Purification and polyclonal antibody production

Inoculating UL45 and UL45Δ positive cloning strain into 5 ml LB/AMP liquid medium respectively and cultivating overnight. 50 μl cultures were inoculated to 5 ml LB/Amp to activation. When the bacterium reached logarithmic phase (at OD600 of 0.5-0.6), adding IPTG (final concentration 0.2 mM) to induce the expression of UL45 and UL45Δ protein. The situation of protein expression was analyzed by SDS-PAGE. The un-induced and vector control culture were analyzed in parallel. To increase the production of the recombinant protein, expression conditions including the temperatures, concentrations of IPTG and durations of induction were optimized.

The UL45Δ protein was purified by IMAC on Ni^2+^-NTA affinity resin and salting-out. The samples from Ni-column and sediments from salting-out were assessed by SDS-PAGE. The purified protein was used to immune New Zealand white rabbits to raise antibody. The antiserum was harvested from the jugular vein and stored at -70°C.

### Purify the antiserum and Western blot analysis

First, the rabbit anti-UL45ΔIgG was precipitated from the polyclonal antiserum by ammonium sulfate precipitation [49]. Then, using a DEAE-Sepharose column, the IgG fraction was purified by ion-exchange column chromatography following the manufacturer's instructions [50]. To characterize the antigenicity of the UL45Δ fusion protein, western blot analysis was performed according to the standard procedure using the purified rabbit anti-UL45Δ IgG and rabbit anti-DEV IgG [51].

### Transcription characteristics analysis of DEV UL45 gene

The DEF was produced by the usual method [47]. When the DEF grew monostratum, inoculated the DEV-CHv. Total cellular RNA was extracted at 0, 0.5, 1, 2, 4, 6, 8, 10, 12, 18, 24, 30, 36, 42, 48, 54, 60, 66 and 72 h post-infection (pi) using the RNAprep Pure Cell/Bacteria Kit. Then the RNA was immediately inversed transcribed to the cDNA by Quant Reverse Transcriptase and stored at -70°C. At the same time detecting the integrality and purity of RNA by electrophoresis on a 3.0% agarose gel and nucleic acid-protein detecting instrument. The fluorescent quantitative real-time PCR (FQ-PCR) primers for UL45 and β-actin (used as the internal parameters, its expression level is relative constancy in cells) were UL45 F (5'- CATGGAGTTGGGTGTGCT -3') and UL45 R (5'-ACGCTGTAGTCGGTATCG -3'), β-actin F (5'-CCGGGCATCGCTGACA-3') and β-actin R (5'- GGATTCATCATACTCCTGCTTGCT-3'). Standard curve of PMD18-T/UL45 and PMD18-T/β-actin (constructed and preserved in the author's laboratory) was established. The transcription kinetics of the DEV UL45 gene during the viral infection was detected by the method of FQ-PCR. The real-time FQ-PCR was performed in an 20 μl reaction mixture containing cDNA 2 μl, SYBR Green I Mix 9 μl, each of the primer 25 pmo1, adding ultrapure water to total volume. Each run consisted of initial denaturation at 95°C for 1 min followed by 40 consecutive cycles of denaturation at 94°C for 30 s, annealing at 58°C for 30 s. Then the fluorescence was measured by 94°C for 60 s and 60°C for 60 s, and then followed by 70 consecutive cycles of 60°C for 10 s, with each cycle increased 0.5°C. β-actin served as the internal parameters done the parallel experiment. Samples and internal parameters were tested in triplicate. The method of 2^-ΔΔCt ^was convenient to measure the relative amount of the UL45 mRNA expression [52].

### Characteristics analysis of UL45 protein

The purified DEV was got from ultra-high speed centrifugation [53,54]. Extracted the purified virion with NP-40 detergent and centrifuged at 70,000 rpm for 60 min at 4°C (Hitachi), and obtaining a supernatant fraction containing the detergent soluble proteins (envelope and minor amounts of some tegument proteins) and a pellet (the nucleocapsids and tegument proteins) [28]. The purified virion and viral ingredients were then analyzed by western blot using the rabbit anti-UL45Δ IgG [51].

## Competing interests

The authors declare that they have no competing interests.

## Authors' contributions

AS and GM carried out most of the experiments and wrote the manuscript. AC and MW critically revised the experiment design and the manuscript. DL, LL, TZ, DZ, QL, RJ, ZC, YZ and XC helped with the experiment. All the authors read and approved the final manuscript.
